# Changing sensitivity to cold weather in Texas power demand

**DOI:** 10.1016/j.isci.2022.104173

**Published:** 2022-03-29

**Authors:** Blake Shaffer, Daniel Quintero, Joshua Rhodes

**Affiliations:** 1 Department of Economics, University of Calgary, Calgary, AB T2N 1N4, Canada; 2 Department of Mechanical Engineering, The University of Texas at Austin, Austin, TX 78712, USA

**Keywords:** Environmental monitoring, Energy policy, Energy engineering, Power engineering

## Abstract

We estimate the effect of heightened temperature sensitivity on electricity demand in Texas during the February 2021 blackout event. Using 20 years of hourly data, we estimate the relationship between temperature and electricity demand; finding demand has become more responsive to cold temperatures over time. This is consistent with the fact electric heating has similarly increased over the past 20 years in Texas. We find during the February 2021 event, average electricity demand was 8% higher, and approximately 10,000 MW higher during the peak hour, than it would have been had temperature sensitivity remained unchanged at early 2000s levels. Our results highlight that Texas’s increased sensitivity to cold weather extremes is not limited to the supply side, but the demand side as well. These findings have implications to other regions that are seeking to reduce carbon emissions through the electrification of heating.

## Highlights

• Texas power demand sensitivity to cold temperature has steepened over the past 20 years
• Heightened sensitivity of demand exacerbated the February 2021 Texas outage event
• Average demand was 8% higher due to greater cold temperature sensitivity
• This has implications for regions that are electrifying heating

## Graphical abstract

**Figure:**
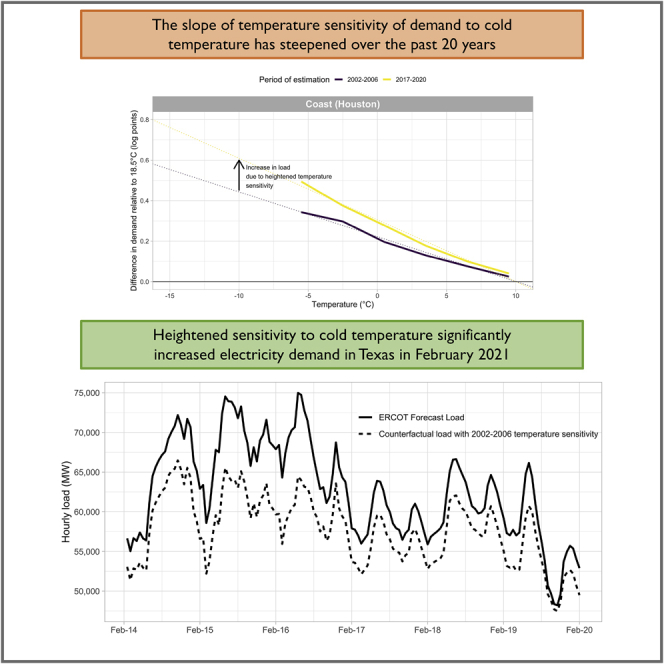

## Introduction

In February 2021, the extreme cold weather from Winter Storm Uri strained the Electric Reliability Council of Texas (ERCOT) power grid to the brink. Over the course of five days, roughly 12 million Texans were left in the dark. In total, nearly 1,000 GWh of firm electric load was shed (not served) as rolling blackouts were enacted to avoid a complete system-wide loss of power. The human health and safety toll was large, with an official estimate of 246 people dying, unprotected in the cold, and ancillary infrastructure, such as water systems, breaking down (Texas Department of State Health Services, 2021). The economic damages from the storm are expected to exceed $100 billion US dollars (Accuweather, 2021).

In the aftermath, much of the focus has been on the supply side of Texas’ power market. The question of which fuel type was “most at fault” became politicized; conversations about the interdependence of electricity and natural gas systems, and the need to winterize both, echoed the same conversations from only ten years ago, during the last “great freeze” in Texas; and ERCOT’s unique “energy-only” market design was called into question as to whether it led to insufficient levels of reliability. All important questions that will undoubtedly be studied and discussed for years to come.

Far less focus, however, has been placed on the demand side. What is clear is just how large electricity demand in the region was, leading up to the blackout event. In ERCOT’s seasonal assessment of resource adequacy for Winter 2020/21, the planned winter peak demand—the basis upon which ERCOT determines whether they have sufficient capacity to meet demand—was 57,699 MW (ERCOT, 2020). An extreme peak load scenario was included in the assessment at 67,208 MW based on a repeat of the 2011 cold weather event in the state. On Sunday February 14, a typically off-peak day, ERCOT set a record peak winter load of 69,692 MW at 8p.m., with forecasts calling for a peak exceeding 76,000 MW for the coming midweek cold snap, before load shed eventually negated such levels from occurring. In terms of daily average load, Sunday February 14th 2021 was the highest daily average load in ERCOT history (see Figure S1).

In this paper, we ask the question: Has electricity demand in Texas become more sensitive to cold weather? And, if so, how much higher was electricity demand during the February event due to heightened temperature sensitivity? We motivate this investigation by noting that over the past 15 years the share of Texan households using electric heat has risen from 52% to 61% (U.S. Census Bureau, 2004; 2019). Given a higher reliance on electricity for heating, it would be reasonable to expect Texas electricity demand to be more sensitive to cold weather events today than in the past. Thus, at the cold temperatures observed in February 2021, even if not the most intensely cold on record (Doss-Gollin et al., 2021), the electrification of heating stands to push electricity demand higher than would previously have been expected.

We answer this question using nearly 20 years of hourly historical weather and electric load data to estimate the temperature responsiveness of electricity demand across each of ERCOT’s eight weather zones, and, importantly, how these temperature response functions have changed over time. In doing so, we are able to calculate the increase in electricity demand during the cold temperatures observed in February 2021 that is attributable to an increasingly cold-weather-responsive demand. We find heightened temperature responsiveness accounts for an average increase in overall ERCOT demand over the outage period of roughly 8% (absent the load shed), and slightly more than 10,000 MW of additional load during the peak hour event on the morning of February 16, than it would have been absent the increase in cold-weather sensitivity. This is after controlling for annual trends, such as population and overall load growth.

These findings highlight Texas’ changing sensitivity to cold weather events. The extreme events in 2021 were not only challenging for the supply side of power generation but also from additional temperature sensitivity to cold weather resulting in greater demand for electricity than would have been the case only a decade ago. We discuss implications to other regions seeing increased electrification of heating as a pathway to net-zero emissions.

Several studies have examined the temperature responsiveness of electricity demand, but largely in the context of increasing temperatures due to climate change (Isaac and van Vuuren, 2009; De Cian and Wing, 2017; Wenz et al., 2017; Auffhammer et al., 2017). Others have considered the adaptive effect of increased air conditioner penetration, leading to heightened responsiveness of electricity demand to higher temperatures (Davis and Gertler, 2015; Rivers and Shaffer, 2020). There is comparatively very little research on the effect of greater electrification of heating in terms of altering the temperature sensitivity of power demand in the domain of cold temperatures. This analysis seeks to fill that knowledge gap.

## Results

### Temperature response functions

**Figure 1:**
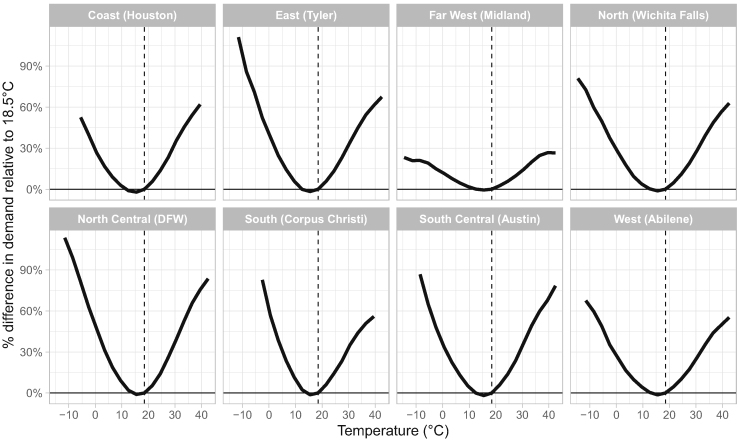
Temperature response functions by ERCOT weather zone; all years Plotted are the estimated regression coefficients representing percentage difference in demand for each respective temperature bin relative to 18.5°C. Note, the coefficients, estimated in log points, have been converted to percentage change by the following: $\beta_{percentchange}=\exp\left(\beta_{estimated}\right)-1$. For small changes, $\beta_{percentchange}\approx \beta_{estimated}$.

We begin by estimating the relationship between temperature and electricity demand, conditioning on other non-temperature factors of demand, as per (Equation 1) in the method details section (below). We do so separately for each ERCOT weather zone, using data from 2002 through 2020. Data from 2021 are excluded from the estimation because (a) we want to avoid the confounding effect of the load shed during the outage event, and (b) the year fixed effect would be problematic given the partial year containing below-average temperatures (i.e. winter period only). We plot the temperature response functions, for each region, in Figure 1. These functions show the expected percentage difference in electricity demand for a given prevailing temperature relative to 18.5°C (approx. 65 °F).

The temperature response functions exhibit their familiar U-shape, consistent with the existing literature, highlighting that electricity demand increases as temperature deviates in both directions from a neutral point, resulting in either heating or cooling load. The steepness of each side of the temperature response function represents the sensitivity to cold and hot temperatures, respectively.

Next, we re-estimate temperature response functions by zone, but this time doing so using separate five-year periods. This allows us to follow the evolution of temperature responsiveness over time. The results are shown in Figure S2 in the supplemental information.

In two of the eight zones, namely the West and Far West, we see a flattening of the temperature response functions over time. It appears load is becoming less sensitive to temperature in these two zones. This is consistent with the continued growth in less temperature-sensitive electrified oil and gas development in west Texas (ERCOT, 2018). A neutral result is observed in the North Zone, with little change over time. Whereas, in the remaining five zones (North Central, Coast, South Central, South, and East), the temperature response functions all steepen in the most recent period of estimation relative to the oldest period, reflecting a heightened sensitivity to cold temperatures. These zones represent the bulk of ERCOT’s load, or roughly 90% of the total, and thus increases in temperature sensitivity in these regions overwhelmingly drive total ERCOT electricity demand.

### Projecting the effect of changing sensitivity to cold temperatures

We now use the estimated temperature response functions to answer the originally proposed question: by how much greater was electric load during the February 2021 event due to heightened cold temperature sensitivity? To do so requires extrapolating the estimated temperature response functions over a range of temperatures outside the domain of temperatures used in the estimation process above.

**Figure 2:**
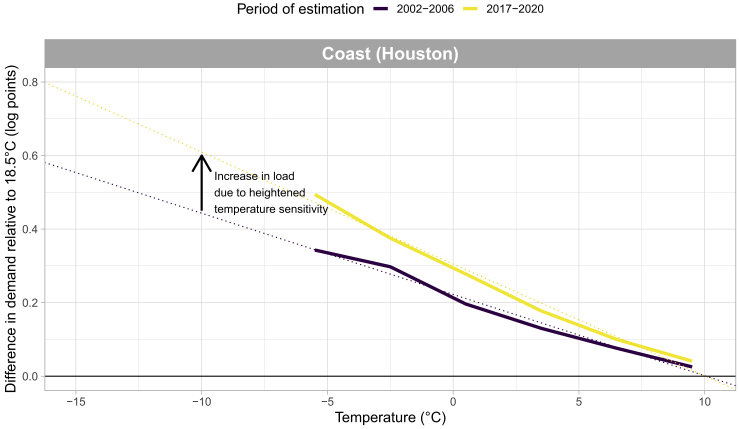
Temperature response function for Coast zone; below 10°C Plotted are the estimated regression coefficients representing percentage difference in demand for each respective temperature bin relative to 18.5°C Note, for visualizing this linear fit, the coefficients are shown in their estimated log points.

Figure 2 plots the temperature response functions for one zone—the Coast Zone—over the domain of cold temperatures (i.e. below 10°C), for the oldest (2002–2006) and newest (2017–2020) periods. From the figure, we see that a linear approximation is a strong fit for the temperature response functions over this range of temperatures, with demand differences measured in log points. Extrapolating this linear fit into the colder temperatures observed in February 2021 allows us to calculate the additional electric load due to heightened temperature sensitivity. The linear assumption has been used by Auffhammer et al. (2017) and Rivers and Shaffer (2020), among others, to extrapolate beyond the domain of estimated temperatures. In those examples, it was for extrapolating to higher temperatures due to climate change. While certainly some non-linearities could exist in the relationship beyond the currently estimated temperature domain, our concern here is the difference between two temperature response functions, and thus it would be *changes in non-linearities over time* that would be the confounding factor.

We repeat the analysis displayed in Figure 2 for all zones. As previously mentioned, in the majority of zones, heightened temperature sensitivity results in more load than would have been the case with 2002–2006 sensitivity; whereas in a few zones, notably smaller ones, the opposite is true. Summing across all zones results in a clear and significant increase in load due to temperature sensitivity.

**Figure 3:**
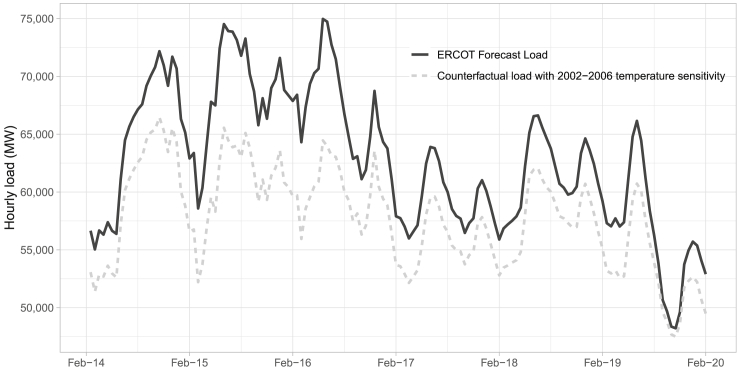
Load forecast and counterfactual load with 2002–2006 temperature sensitivity ERCOT forecast load is used in place of actual load as the firm load shed truncated desired demand. The counterfactual hourly load is calculated by taking the ERCOT load forecast and adding the difference between the estimated load deviation based on 2002–06 temperature responsiveness and 2017–20 responsiveness, both evaluated at the observed hourly temperatures in February 2021.

Figure 3 plots hourly total ERCOT load over the period of February 14–19, 2021. The actual ERCOT forecast load is represented by the solid line. This is the load forecast by ERCOT had there not been firm load shed (i.e. blackout) events. The dashed line is the estimated counterfactual load had ERCOT had the temperature sensitivity observed in 2002–2006 as compared to 2017–2020. It is constructed by taking the ERCOT load forecast and adding the difference between the estimated load deviation based on 2002–06 temperature responsiveness and 2017–20 responsiveness, both evaluated at the observed hourly temperatures in February 2021.

At the peak, during the morning of February 16, our model indicates heightened temperature responsiveness accounts for an additional 10,800 MW of demand. Across the entire period of the February 2021 blackouts, we estimate heightened temperature sensitivity increases total forecast load by 8%, as compared to what load would have been had temperature sensitivity remained at 2002–06 levels. This effect is after controlling for annual trends, such as population growth, and is purely indicative of increased temperature sensitivity.

## Discussion

Temperature sensitivity to cold weather in most parts of Texas has increased over the past 20 years. We estimate this increased sensitivity resulted in an additional 8% average load during the extreme cold of February 2021 than would have occurred had temperature responsiveness remained at 2002–06 levels. At the same time, electric heating shares have risen from 52% to 61% of households, an increase of roughly two million homes, in the state of Texas over the past 15 years (U.S. Census Bureau, 2004; 2019). We stress that lack of data preclude us from making causal claims as to the link between electric heating and the observed change in sensitivity—the relationship we observe is strictly correlative—however, our results are consistent with results from a simulation study (White and Rhodes, 2019) that shows increased electric heating in the Texas’s residential sector leading to larger winter electricity peaks.

Our results highlight Texas’s heightened susceptibility to extreme cold weather events, a fact much discussed on the supply side of the power market, but with little conversation as to shifts in demand. The prevalence of inefficient electric resistance heat may play a large role in Texas’s temperature responsiveness to cold weather; a shift toward more efficient heat pumps could prove valuable in reducing energy needs during cold weather events, though at extreme temperatures these efficiency gains may be reduced. Our findings suggest more work on the direct link between changes to heating equipment and electricity demand should be undertaken to improve the accuracy of load forecasts to incorporate changing relationships.

Our results also have broader implications to other regions, in the United States and globally, increasing their electrification of space heating. As efforts to achieve net-zero emissions increase, and electrification of space heating plays a central element in many pathways, this paper emphasizes the need to incorporate the increased prevalence of electric heating to rethink temperature-demand relationships. Heightened temperature sensitivity will require greater capacity in cold weather conditions than previously considered to ensure electric reliability.

### Limitations of the study

This study provides a historical correlative analysis of the relationship between cold weather and electricity demand in Texas, and how it has changed over time. Two limitations of the current analysis are (1) its usefulness for prediction, and (2) its lack of causal link as to the underlying mechanism that is changing the relationship between cold temperatures and electricity demand. We discuss each in turn.

#### Prediction

We control for changes over time using a year fixed effects approach. This approach means we do not need to arbitrarily specify the factors that affect demand over time, allowing the year fixed effect to flexibly subsume all potential factors. However, it limits our study’s usefulness for prediction purposes. An alternative approach would be to include a large set of conditioning variables, such as population growth, GDP, industrial composition, etc., allowing for predictions of future demand by incorporating assumptions on the future level of these variables. The trade-off with such an approach is potential omitted-variable bias and the requirement of assumptions around the value of these conditioning variables for prediction.

#### Causal mechanism

We highlight the associative link between the increased sensitivity of electricity demand to cold weather and the increased use of electric space heating in Texas. However, this link is purely correlative, as we have insufficiently granular data to perform a proper causal analysis using panel data. A potentially fruitful area of future research would be to use household-level electricity data, along with a household-level panel on space heating, to properly estimate the causal link between electric space heating adoption, cold temperature, and electricity demand.

## STAR★Methods

### Key resources table

**Table:** 

RESOURCE	SOURCE	IDENTIFIER
**Deposited data**		

Weather Data	National Oceanic and Atmospheric Administration (NOAA)	https://www.ncdc.noaa.gov/cdo-web/datatools/lcd/
Electric Load Data	Electric Reliability Councilof Texas (ERCOT)	http://www.ercot.com/gridinfo/load/load_hist/
Heating Type Data	United States Census Bureau	https://data.census.gov/cedsci/

**Software**		

R	R for Statistical Computing	https://www.r-project.org

### Resource availability

#### Lead contact

Further information and requests for comment should be directed to and will be fulfilled by the lead contact, Blake Shaffer (blake.shaffer@ucalgary.ca).

#### Materials availability

Not applicable.

### Method details

In this method details section, we first describe the key data sources used in the analysis and present summary statistics. This is followed by a detailed description of our temperature response function estimation method.

#### Temperature response function estimation method

We estimate the temperature response function, i.e. the relationship between temperature and electricity demand, using hourly load and temperature data, separately across each ERCOT Weather Zone. Specifically, we run eight separate regressions, one for each Zone (z), regressing the logarithm of hourly load ($y_{t}$) on hourly temperature variables ($T_{tb}$) and a rich set of date and time fixed effects ($X_{t}$):

(Equation 1) $$\text{log}\left(y_{t;z}\right)=\sum_{b}\beta_{b;z}T_{bt;z}+\theta_{z}X_{t;z}+\epsilon_{t;z},\;\forall z$$

For the temperature variables, $T_{btz}$, we use binned temperature dummies in 3°C increments, across the range of observed temperatures in Texas from 2002 through 2021. For example, the variable $T_{8-11{}^{\circ}C,tz}$ receives a ‘1’ if the temperature in hour *t* in zone *z* falls in bin *b* = (8 to 11°C], and a ‘0’ otherwise. The omitted bin is the one centred around 18.5°C (roughly 65 °F), and thus the interpretation of each bin’s coefficient is the log difference in demand between temperatures in the respective bin as compared to demand when temperature is 18.5°C. For small changes, the log difference in demand can be roughly interpreted as the percentage change in demand, i.e. 0.1 ≈ 10% change. This approach allows for a flexible relationship between temperature and demand. An alternative approach, also used in the literature, is to use heating and cooling degree days, essentially the absolute difference between recorded temperature and a “neutral” baseline of 18.5°C. We use temperature bins as it avoids the requirement to impose an arbitrary nadir to the non-linear temperature-demand relationship. Any temperature bins with less than 10 hourly observations are dropped due to imprecision of their estimates.

$X_{t}$ is a vector of date and time fixed effects that are predictable factors of demand. These include dummy variables for year, month, day of week, and hour of day. Year fixed effects control for annual load growth trends. This flexibly controls for levels shifts in demand due to time-varying factors, such as population and prices. As a robustness check, we also run regressions using population-normalized demand as the dependent variable. We do so by taking the logarithm of demand per capita in kWh, using hourly load data and annual county-level population data aggregated to the ERCOT weather zone. While year fixed effects remove level shifts in average demand due to population growth and other factors, population-normalizing the demand variable allows us to check if sensitivity differs more or less in extreme conditions due to population changes. We find no significant difference in the results. Monthly fixed effects control for predictable seasonality in electricity demand independent of weather. Day of week controls for typical weekday/weekend fluctuations in demand, and hour of day controls for predictable patterns of the intraday shape of demand. We include interactions between month and hour, and between day of week and hour, to reflect that the hourly demand profile differs both seasonally and across different days of the week. Our identifying assumption is that after conditioning on these predictable factors of demand, the variation in shocks to electricity demand ($\varepsilon_{t}$) are uncorrelated with temperature. Because of the high resolution of fixed effects covering key drivers of electricity demand that we include in our specification, as well as year fixed effects making our identification based on within-year variation, we believe that this specification should successfully identify the short-run effect of temperature on consumption. We note, also, that this method follows that of many papers in the existing peer-reviewed literature (Auffhammer et al., 2017; Wenz et al., 2017; Rivers and Shaffer, 2020).

After performing this estimation separately for each zone over all years in the dataset, we then separately estimate the temperature response functions using data by zone in 5 year increments to determine the evolution of temperature response functions over time. Specifically, a steepening of the left hand side of the temperature response function—the region of cold temperatures—indicates heightened cold temperature sensitivity and thus for comparable cold temperatures, electricity demand is expected to be higher, all else equal.

## Data Availability

• All data used in the analysis (electric load, weather, and heating type) have been deposited at https://github.com/blakeshaffer/ercotproject/ and are publicly available as of the date of publication. • All original code used in the analysis has been deposited at https://github.com/blakeshaffer/ercotproject/ and is publicly available as of the date of publication. • Any additional information required to reanalyze the data reported in this paper is available from the lead contact upon request.
