# Supplementary material for: Changing sensitivity to cold weather in Texas power demand
Source: iScience. 2022 Mar 29;25(4):104173. doi: 10.1016/j.isci.2022.104173 (PMC9010639; doi:10.1016/j.isci.2022.104173)
Supplement: Document S1. Figures S1–S3 and Table S1 [file mmc1.pdf]

iScience, Volume *25*

Supplemental information

Changing sensitivity to cold weather in Texas power demand

Blake Shaffer, Daniel Quintero, and Joshua Rhodes

Supplementary Information

Electric load data

We estimate the relationship between temperature and electricity demand using hourly data from 2002 through 2021 across each of ERCOT's eight weather zones, shown in Figure S1. The electric load data come directly from ERCOT's hourly load data archives (ERCOT, 2021) and are summarized in Table S1. The North Central, Coast, and South Central zones, comprising the major cities of Dallas-Fort Worth, Houston, and Austin, are significantly larger than the rest, representing nearly 80% of total load in ERCOT.

Figure S1: ERCOT Weather Zone Map

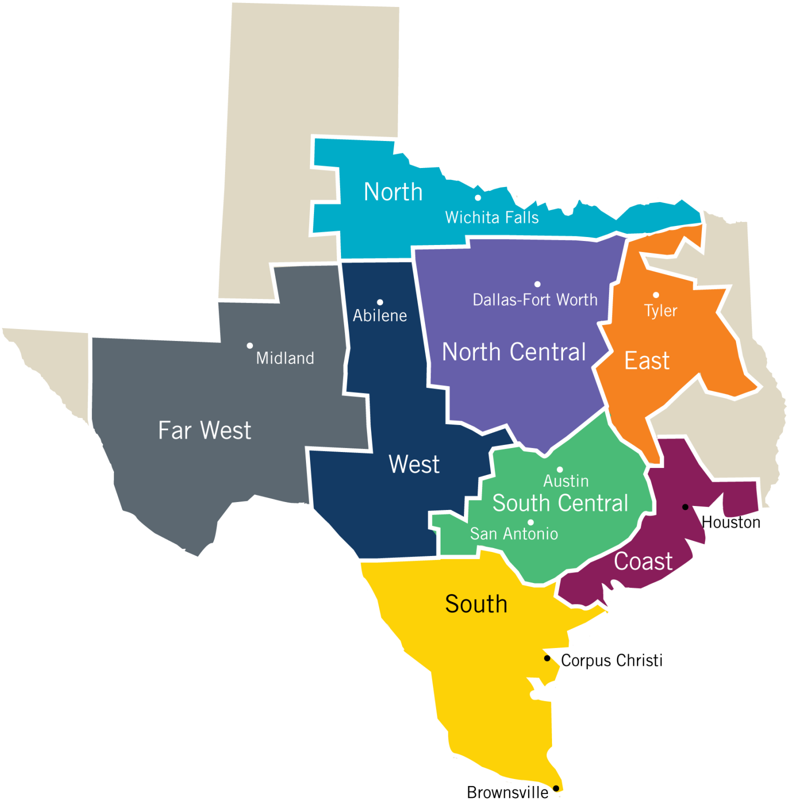

Data source: ERCOT.com

Table S1: Summary statistics for Texas electric load and temperature, 2002-2021

	**Load (MW)**			**Temp (°C)**		
**Weather Zone**	Mean	Max	Min	Mean	Max	Min
Coast (Houston)	10754	21256	1703	21	42	-11
East (Tyler)	1393	2949	709	19	43	-18
Far West (Midland)	1802	4439	843	18	45	-19
North (Wichita Falls)	941	2306	488	18	44	-21
North Central (DFW)	12413	26499	5819	20	43	-18
South (Corpus Christi)	2951	6192	1330	22	41	-8
South Central (Austin)	6142	14167	2858	20	43	-14
West (Abilene)	1048	2117	502	18	43	-20

Data sources: Electric load data from ERCOT.com. Temperature data from airport weather stations from NOAA LCD. Location of major population weather station within each ERCOT zone shown in parentheses. Period January 2002 through February 2021.

Weather data

The weather data are collected from NOAA Local Climatological Data (LCD) (NOAA, 2021). We use the variable 'dry bulb temperature', which is the moisture-free measurement of ambient air temperature. We select data from the major city for each respective zone, as shown in Figure S1, as being reflective of weather conditions for the zone in our temperature response estimation. For the South Central and South zones, which list multiple cities in Figure S1, we select Austin and Corpus Christi, respectively. The weather data are summarized in Table S1.

Heating type data

The heating type data are collected from the U.S. Census Bureau's 2004 and 2019 American Community Survey (ACS), in the Public Use Microdata Sample (PUMS), and selecting the variable 'House Heating Fuel' (U.S. Census Bureau, 2019, 2004). This variable includes 1-year estimates of households using various fuel types for main heating, from which we compute shares by fuel type for all households in Texas. The share of households using electricity for heating rises by 9%, from 52% to 61%, while the share of households using natural gas declines by 7%, going from 42% to 35% over this 15 year period.

Additional figures

Figure S2: ERCOT Average Daily Electricity Demand, 2002-2021

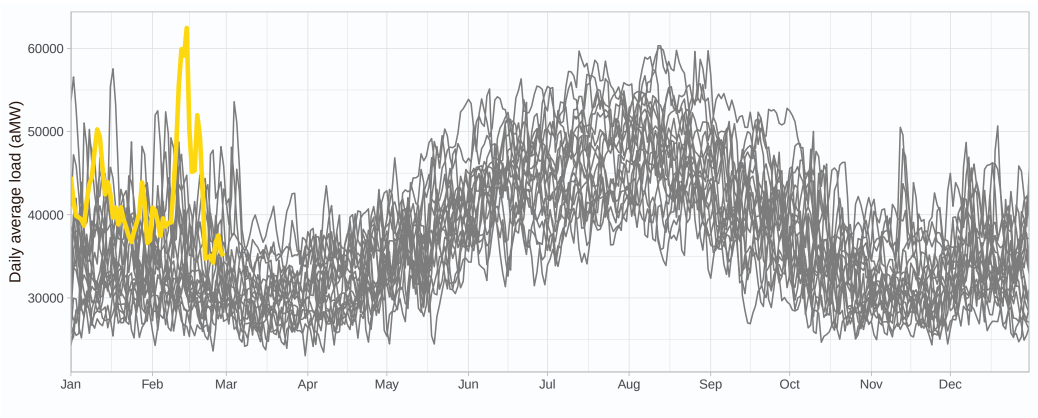

Data source: ERCOT.com; calculations by Authors. 2021 shown in yellow.

Figure S3: Temperature Response Functions by ERCOT Weather Zone; in 5 year increments

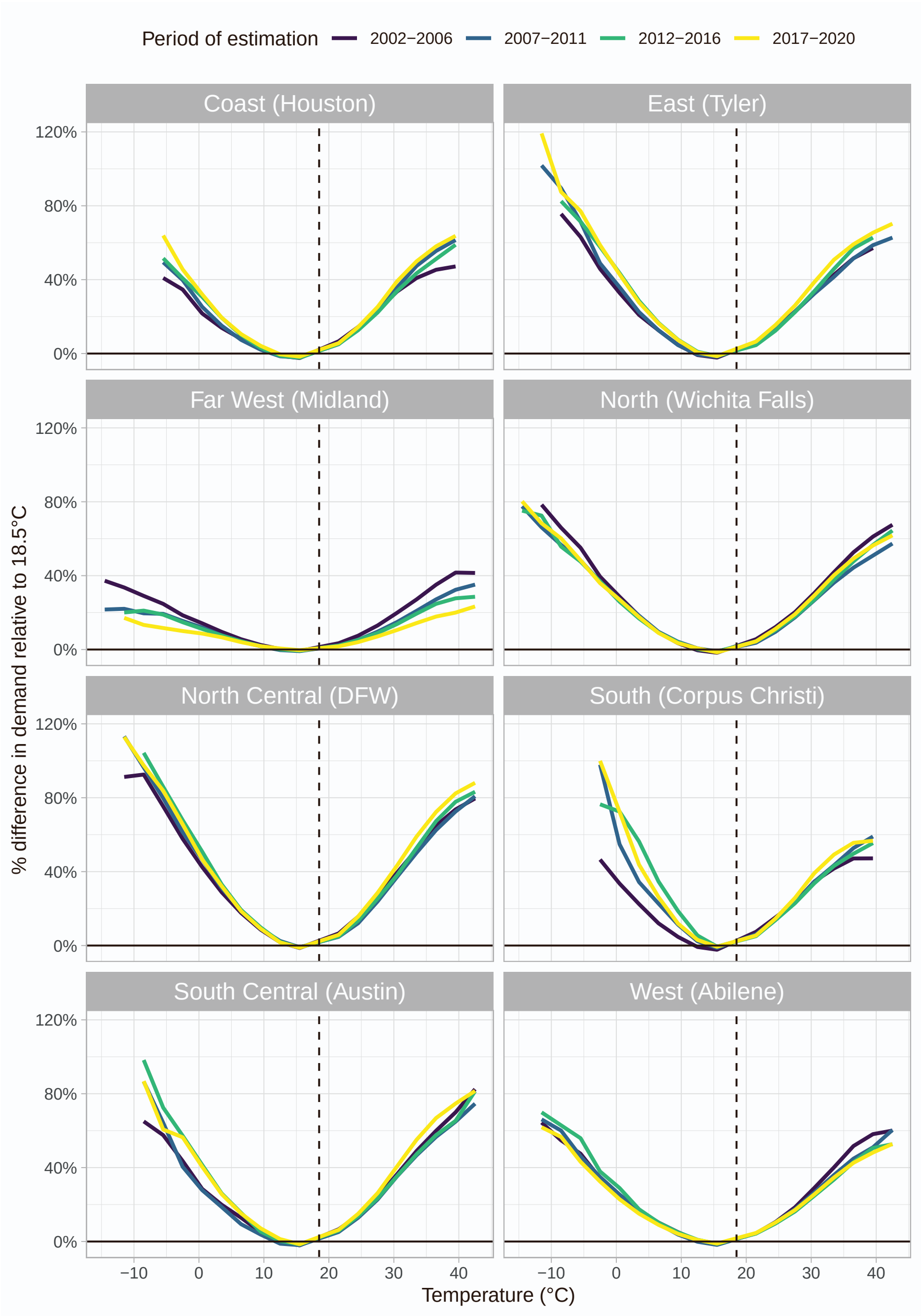

Figure notes: Plotted are the estimated regression coefficients representing percentage difference in demand for each respective temperature bin relative to 18.5°C. Note, the coefficients, estimated in log points, have been converted to percentage change by the following: $\beta_{percentchange} = exp(\beta_{estimated}) - 1$. The temperature response functions are estimated separately in 5 year increments of the data.

References

ERCOT, 2021. Hourly Load Data Archives. http://www.ercot.com/gridinfo/load/load_hist/. [Online; accessed 17-March-2021].

NOAA, 2021. Local Climatological Data. https://www.ncdc.noaa.gov/cdo-web/datatools/lcd/. [Online; accessed 17-March-2021].

U.S. Census Bureau, 2004. 2004 American Community Survey. https://data.census.gov/mdat/#/search?ds=ACSPUMS1Y2004&rv=ucgid,HFL&wt=WGTP&g=0400000US48. [Online; accessed 17-March-2021].

U.S. Census Bureau, 2019. 2019 American Community Survey. https://data.census.gov/mdat/#/search?ds=ACSPUMS1Y2019&rv=ucgid,HFL&wt=WGTP&g=0400000US48. [Online; accessed 17-March-2021].
